# Proteomic Analysis of Ubiquitinated Proteins in Rice (*Oryza sativa*) After Treatment With Pathogen-Associated Molecular Pattern (PAMP) Elicitors

**DOI:** 10.3389/fpls.2018.01064

**Published:** 2018-07-23

**Authors:** Xiao-Lin Chen, Xin Xie, Liye Wu, Caiyun Liu, Lirong Zeng, Xueping Zhou, Feng Luo, Guo-Liang Wang, Wende Liu

**Affiliations:** ^1^State Key Laboratory for Biology of Plant Diseases and Insect Pests, Institute of Plant Protection, Chinese Academy of Agricultural Sciences, Beijing, China; ^2^The Provincial Key Lab of Plant Pathology of Hubei Province, College of Plant Science and Technology, Huazhong Agricultural University, Wuhan, China; ^3^Department of Plant Pathology, Center for Plant Science Innovation, University of Nebraska, Lincoln, NE, United States; ^4^School of Computing, Clemson University, Clemson, SC, United States; ^5^Department of Plant Pathology, Ohio State University, Columbus, OH, United States

**Keywords:** elicitors, hormone signaling, phenylpropanoid pathway, plant defense, quantitative proteomics, ubiquitination

## Abstract

Reversible protein ubiquitination plays essential roles in regulating cellular processes. Although many reports have described the functions of ubiquitination in plant defense responses, few have focused on global changes in the ubiquitome. To better understand the regulatory roles of ubiquitination in rice pattern-triggered immunity (PTI), we investigated the ubiquitome of rice seedlings after treatment with two pathogen-associated molecular patterns, the fungal-derived chitin or the bacterial-derived flg22, using label-free quantitative proteomics. In chitin-treated samples, 144 and 167 lysine-ubiquitination sites in 121 and 162 proteins showed increased and decreased ubiquitination, respectively. In flg22-treated samples, 151 and 179 lysine-ubiquitination sites in 118 and 166 proteins showed increased and decreased ubiquitination, respectively. Bioinformatic analyses indicated diverse regulatory roles of these proteins. The ubiquitination levels of many proteins involved in the ubiquitination system, protein transportation, ligand recognition, membrane trafficking, and redox reactions were significantly changed in response to the elicitor treatments. Notably, the ubiquitination levels of many enzymes in the phenylpropanoid metabolic pathway were up-regulated, indicating that this pathway is tightly regulated by ubiquitination during rice PTI. Additionally, the ubiquitination levels of some key components in plant hormone signaling pathways were up- or down-regulated, suggesting that ubiquitination may fine-tune hormone pathways for defense responses. Our results demonstrated that ubiquitination, by targeting a wide range of proteins for degradation or stabilization, has a widespread role in modulating PTI in rice. The large pool of ubiquitination targets will serve as a valuable resource for understanding how the ubiquitination system regulates defense responses to pathogen attack.

## Introduction

Plants have developed sophisticated mechanisms to defend themselves against pathogen infection. As an example, plants recognize pathogen-associated molecular patterns (PAMPs) through plasma membrane-located pattern recognition receptors (PRRs), and transduce the perceived signal to downstream components, including mitogen-activated protein kinase (MAPK) cascades in the cytoplasm. This process culminates in the activation of pattern-triggered immunity (PTI). To overcome PTI, pathogens can secrete effector proteins into host plant cells to suppress or avoid PTI. However, as a countermeasure, plants have evolved cytoplasmic immune receptors that recognize the pathogen-derived effectors and activate a robust second layer of plant immunity, called effector-triggered immunity (ETI) (Jones and Dangl, 2006).

The interactions of receptors of PAMPs, such as the chitin receptor CERK1 or the flg22 receptor FLS2, have been extensively studied in *Arabidopsis thaliana* (Gómez-Gómez and Boller, 2000; Miya et al., 2007; Macho and Zipfel, 2014). Although early molecular events associated with different receptor – PAMP interactions are distinct, the subsequent gene expression patterns extensively overlap, and PTI- and ETI-mediated changes in gene expression are also broadly similar (Boller and Felix, 2009). Both PTI and ETI involve various signaling pathways and cellular responses mediated by plant hormones, such as the salicylic acid (SA), jasmonic acid (JA), and ethylene (ET) (Broekaert et al., 2006; Loake and Grant, 2007; Balbi and Devoto, 2008). The downstream cellular responses of PTI and ETI include production of reactive oxygen species (ROS) (Frederickson Matika and Loake, 2014), activation of pathogenesis-related (PR) gene expression (Sels et al., 2008), and accumulation of secondary metabolites (Piasecka et al., 2015). ROS are rapidly produced for cell signaling and defense, and especially during the hypersensitive response (HR). Because excessive ROS is harmful for plant cells, various ROS-detoxifying enzymes also accumulate during defense responses to maintain the redox balance (Frederickson Matika and Loake, 2014). PR genes, such as those encoding chitinases, are induced to suppress pathogen invasion (Sels et al., 2008), while secondary metabolites, such as the phenolic compounds flavonoids and lignin precursors, are also important components of defense responses and in some cases can scavenge ROS (Piasecka et al., 2015).

Ubiquitination is a key mechanism for the regulation of plant immunity (Marino et al., 2012) and of many other physiological processes in plants, such as the activation of receptors and their trafficking (Furlan et al., 2012), phytohormone signaling (Kelley and Estelle, 2012), and responses to abiotic or biotic stresses (Zeng et al., 2006; Zhang et al., 2015). Indeed, many proteins involved in plant immune responses, such as plasma membrane receptors, have been identified as ubiquitination targets (Wang et al., 2006; Gimenez-Ibanez et al., 2009; Lu et al., 2011). Studies of the significance of ubiquitination in plant immunity have focused on E3 ligase proteins (Duplan and Rivas, 2014; Zhou and Zeng, 2017). For example, the SCF complex-interacting protein, SGT1, which was the first identified component of E3 ligase, was shown to play a role in defense responses (Azevedo et al., 2002). Other E3 ligases, including OsCOI1b, GID2, and OsCUL3a, also contribute to defense responses. OsCOI1b can interact with JAZ proteins and target them for degradation in response to JA signaling (Yang et al., 2012). GID2, which is an F-box subunit of the SCF E3 complex, specifically interacts with phosphorylated SLR1 protein and regulates the gibberellin-dependent degradation of SLR1 in rice (Gomi et al., 2004). As a component of the Cullin3-based RING E3 ubiquitin ligases, OsCUL3a negatively regulates cell death and immunity by degrading OsNPR1 in rice (Liu et al., 2017). In contrast, the number of known substrate proteins targeted by ubiquitination is very limited, and little is known about how ubiquitination fine-tunes plant defense responses.

Different methods have been used for the proteomic analysis of ubiquitination in *Arabidopsis* (Maor et al., 2007; Manzano et al., 2008; Saracco et al., 2009; Kim D.Y. et al., 2013; Svozil et al., 2014). For example, approximately 950 ubiquitination substrates were identified in seedlings treated with the proteasome inhibitor MG132 (Kim D.Y. et al., 2013), and 1791 ubiquitinated substrates were identified in leaves and roots treated with the more specific proteasome inhibitor, Syringolin A (SylA) (Svozil et al., 2014). These ubiquitination substrates are associated with various cellular processes, such as transport, signal transduction, transcription, translation, and metabolism. Our previous study identified 464 ubiquitinated proteins in young rice leaves through affinity purification and high resolution liquid chromatography tandem mass spectrometry (LC–MS/MS) analysis (Xie et al., 2015). Quantitative proteomic analysis also revealed changes in ubiquitination levels at different plant development stages or in response to different stresses. Under high salt stress, for example, a number of differentially expressed ubiquitinated proteins were identified in rice roots (Liu et al., 2012), while in petunia, a model plant for flower senescence studies, nearly 500 proteins were found to have significantly changed ubiquitination levels upon ethylene treatment (Guo et al., 2017).

In this study, we investigated potential changes in ubiquitination status upon activation of rice defense responses and generated an in-depth quantitative proteomic catalog of ubiquitination in rice seedlings after chitin and flg22 treatments. Through a combination of highly sensitive immune affinity purification, LC–MS/MS, and protein functional classification analysis, we identified 1,376 ubiquitinated proteins, of which 100s were found to show significant changes in their level of ubiquitination. Further analyses demonstrated that the ubiquitination system can coordinate different hormone signaling pathways and diverse cellular processes, including the phenylpropanoid metabolic pathway, as part of a defense response. Our data provides new insights into the function of the ubiquitination system in rice PTI.

## Materials and Methods

### Sample Preparation

Rice (Japonica cultivar Nipponbare) was used in this study. Rice seeds were cultivated in ½ strength Murashige and Skoog medium at 28°C for 1 week. Rice seedlings were immersed in 8 nmol/L chitin (Macklin, China) or 1 μmol/L flg22 (Santa Cruz, CA, United States) solution for 0, 0.5, 1, 3, 6, and 12 h. Water-treated rice seedlings were used as controls. Three biological replicates were used for assessment.

As a source of protein extracts, the rice seedlings were frozen in liquid nitrogen and finely ground to powders in liquid nitrogen. A 5× volume of TCA (trichloroacetic acid)/acetone (1:9) was added to the powder and mixed by vortexing. After storage at -20°C for 4 h, the mixture was centrifuged at 6,000 *g* for 40 min at 4°C; the supernatant was subsequently discarded, and the pellet was washed for three times with cold acetone. About a 30× volume of potassium dihydrogen phosphate buffer was added to the air dried pellet. The sample was sonicated, boiled for 15 min, and centrifuged at 14,000 *g* for 40 min. The supernatant passed through a 0.22 μm filter. The protein concentration of each sample was quantified using the BCA Protein Assay Kit (Bio-Rad, United States). A 20 μg quantity of protein from each sample was fractionated on a 12% SDS-PAGE gel, and the protein bands were stained with Coomassie Blue R-250 (Thermo Scientific, United States).

### Quantitative Reverse Transcription PCR (qRT-PCR)

RNA samples extracted from rice seedlings 3 h after water, chitin, or flg22 treatment were extracted using TRIzol reagent (Invitrogen) and were treated with DNaseI (Invitrogen) to remove genomic DNA. 1 mg of total RNA was used to synthesize the first strand of cDNA using the Reverse Transcription System (Promega) according to manufacturer’s instructions. Quantitative PCR reaction system was prepared using SYBR Green Supermix (Bio-Rad) solution, and qRT-PCR reaction was performed on an ImyiQ2 real-time PCR detection system (Bio-Rad). Data analysis was performed using IQ5 software (Bio-Rad). Expression of defense-related genes were normalized by comparing with a housekeeping gene *OsUBQ1*. Expression of the defense-related genes after chitin or flg22 treatment for 3 h were compared to that after water treatment for 3 h. Gene-specific primers used in this experiment were listed in Supplementary Table S9.

### Affinity Enrichment of Ubiquitinated Peptides

About 10 mg of protein from each sample was used for trypsin digestion and affinity enrichment. Dithiothreitol (DTT) was added to the protein extracts from each sample to a final concentration of 10 mM, and the samples were incubated on an orbital shaker (600 rpm, 37°C) for 1.5 h. After the samples had cooled to room temperature, iodoacetamide (IAA) was added to a final concentration of 50 mM, and the samples were incubated for 30 min in the dark. Four volumes of 50 mM Tris-HCl (pH 8.0) were added to dilute the urea concentration to 2 mM before 200 μg of trypsin (Promega, Madison, WI, United States) was added, and the samples were incubated overnight at 37°C. Trifluoroacetic acid (TFA) was then added to a final concentration of 0.1% in order to reduce the pH to ≤3. C18 Cartridges (Empore^TM^ SPE Cartridges C18, bed I.D. 7 mm, volume 3 ml, Sigma, United States) were used to desalt the samples, which were then concentrated by vacuum centrifugation.

The resulting lyophilized peptides were dissolved in immunoaffinity purification (IAP) buffer (50 mM MOPS-NaOH, pH 7.2, 10 mM Na_2_HPO_4_, and 50 mM NaCl), and the samples were centrifuged at 10,000 *g* at 4°C for 10 min. For each sample, 250 μg of di-Gly-Lys antibody cross-linked to agarose beads was used (PTM Scan ubiquitin remnant motif K-𝜀-GG kit, Cell Signaling Technology, United States), and di-Gly-Lys-containing peptides were enriched as previously described (Xie et al., 2015).

### Mass Spectrometry (MS)

The peptide mixture was loaded onto a reverse phase trap column (Acclaim PepMap100, Thermo Scientific, United States), which was connected to a C18-reversed phase analytical Thermo Scientific^TM^ Easy Column (Thermo Scientific, United States) in buffer A (0.1% formic acid). The peptide mixture was then separated with a linear gradient of buffer B (84% acetonitrile and 0.1% formic acid) at a flow rate of 300 nL/min. LC–MS/MS analysis was performed using a Q Exactive mass spectrometer (Thermo Scientific, United States) and a Proxeon Biosystems Easy nLC^TM^ system (Thermo Scientific, United States). The positive ion mode was used, and data were acquired using a data-dependent top10 method, which dynamically selects the most abundant precursor ions from the survey scan for higher energy collisional dissociation (HCD) fragmentation. For the MS analysis, the following parameters were set: the automatic gain control (AGC) target was set to 3e6, the maximum injection time was 10 ms, dynamic exclusion duration was 40.0 s, survey scan resolution was 70,000, resolution for HCD spectra was 17,500, and isolation width was 2 m/z. Normalized collision energy was 30 eV, and the underfill ratio was 0.1%.

### Database Search and Quantification

The proteins and their ubiquitination sites were identified using the Andromeda search engine in Max Quant (version 1.3.0.5) (Tyanova et al., 2016). The tandem MS data were used to query the MSU Rice Genome Database^1^. The MS/MS tolerance was set at 0.1 Da, and the precursor mass tolerance was set at 6 ppm. Trypsin/P was specified as the enzyme, and four missed cleavages were permitted. During the database search, the fixed modification was carbamidomethylation for cysteines, the variable modifications were Gly-Gly modification for lysines, the false discovery rate (FDR) threshold for modification sites was 0.01, the minimum peptide length was 5, and the FDR was <0.01. Label-free quantification was carried out using MaxQuant as previously described (Tyanova et al., 2016). Precursor ion intensity of modified peptides was used to quantify each peptide. Quantification was based on direct mass spectrometric signal intensities of the given peptides. The differences between the groups were assessed using a *t*-test with Excel 2010 software (Microsoft, United States). *P*-values were subsequently calculated from the *t*-test. The results were considered statistically significant at *P* < 0.05. No imputation was used to treat missing values. The missing values were not filed or replaced, and only the data with at least two values of the three replicates can be used to perform a *t*-test. A 2.0-fold cut-off was used to determine quantitative changes of up-regulated and down-regulated ubiquitinated proteins, with a *P* < 0.05.

### Bioinformatic Analysis

The Gene Ontology (GO) annotation of the ubiquitination proteome was derived from the UniProt-GOA database^2^. The lysine ubiquitination peptide ID was mapped to a GO ID after conversion to a UniProt ID. The ubiquitinated proteins were classified by GO annotation based on the “biological processes,” “molecular functions,” and “cellular components” categories. The *P*-value that applied a hypergeometric distribution with FDR correction was used for calculation to obtain significant enrichment GO catalogs, and the GO terms with *P* < 0.05 were considered to be enriched. The Kyoto Encyclopedia of Genes and Genomes (KEGG) database was used to annotate the protein pathways. KEGG terms with *P* < 0.05 were considered to be enriched. The online service tool KAAS was used to annotate the KEGG database description of the ubiquitinated proteins^3^. The resulting annotations were mapped onto the KEGG pathway database using KEGG Mapper. Protein interaction networks were generated using the STRING database^4^ and were visualized by Cytoscape software, version 3.4.0. (Shannon et al., 2003).

### Western Blot Analysis

To examine ubiquitination levels, proteins were extracted from rice seedlings that had been treated with water, 8 nmol/L chitin, or 1 μmol/L flg22. Samples were collected at different time points. Total proteins were extracted by grinding samples in protein extraction buffer [50 mM Tris-HCl (pH 7.5), 150 mM NaCl, 10 mM MgCl_2_, 1 mM EDTA, 10% glycerol, 0.1% Nonidet P-40, and protease inhibitor cocktail (Sigma Aldrich, United States)]. Total proteins were separated on 10% SDS-PAGE gel and transferred onto a polyvinylidene difluoride (PVDF) membrane (Immobilon-P, Merck Millipore, United States). Ubiquitination levels were then determined by immunoblotting using anti-ubiquitin as an primary antibody (1:5,000, PTM BIO, China) and anti-rabbit secondary antibody conjugated to HRP (1:10,000, PTM BIO, China). Total proteins were evaluated by using anti-HSP antibody (Beijing Protein Innovation, China). The chemiluminescence signal was detected with an X-ray film (Thermo Fisher, United States).

## Results

### Strategy for Quantitative Analysis of the Rice Ubiquitome

**FIGURE 1:**
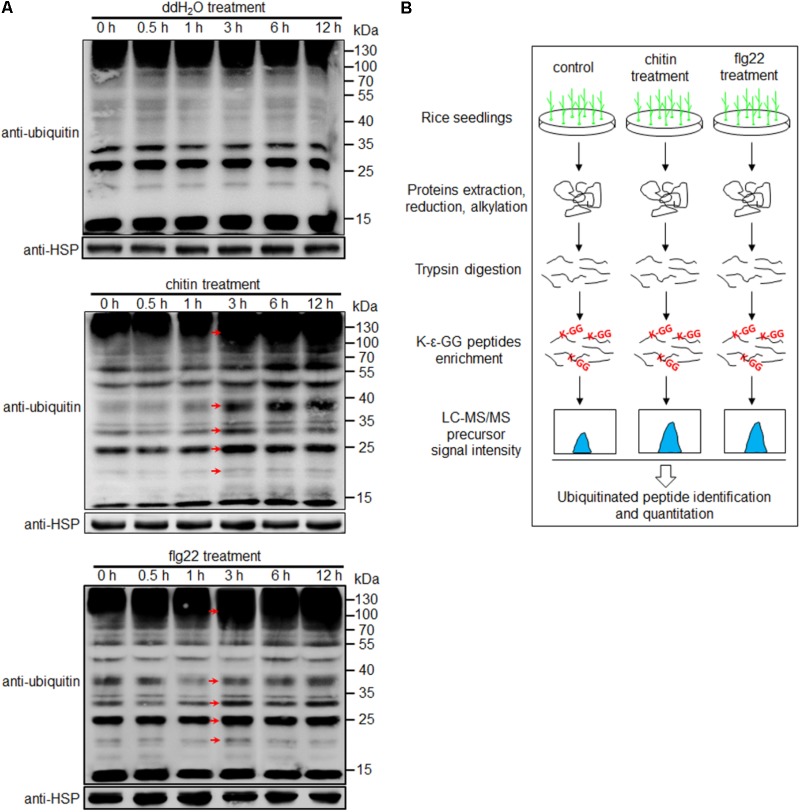
Workflow for quantitative profiling of the ubiquitome. **(A)** Western blot analysis using an anti-ubiquitin antibody. Protein extracts (20 μg) were separated by 12% SDS-PAGE and subjected to Western blot analysis. An anti-HSP antibody was used as a loading control. Arrows indicate signals enhanced at 3 h after treatment with water, chitin or flg22 compared with the 0, 0.5, and 1 h time points. **(B)** Workflow for quantitative profiling of the ubiquitome in 1-week-old rice seedlings treated with chitin or flg22.

To examine the diversity and relative abundance of ubiquitinated proteins induced by PAMPs, we immersed 1-week-old rice seedlings in chitin (8 nmol/L) or flg22 (1 μmol/L) solution for 0, 0.5, 1, 3, 6, and 12 h. Proteins extracted from the treated samples were subjected to Western blot analysis, using an anti-ubiquitin antibody. We observed that proteins with a wide range of molecular masses were ubiquitinated, and a strong immunoblot signal was observed at 3 h after both treatments; no changes were evident in the water-treated seedlings (**Figure 1A** and Supplementary Figure S1). It is worth to note that although the same amount of total protein had been used in the blots, a slightly stronger immunoblot signal were observed in chitin- or flg22-treated than water-treated sample at 0 h time point (**Figure 1A**), the differences might be due to the variations in western blottings. To determine whether chitin or flg22 can induce defense responses in 1-week-old rice seedlings at 3 h, we detected the expression of the known PTI-activated genes in rice seedlings treated by chitin or flg22 using quantitative real-time PCR (qRT-PCR). Expression of the defense-related genes after chitin or flg22 treatment for 3 h (relative to *OsUBQ1*) were compared to their expressions after water treatment for 3 h (relative to *OsUBQ1*). Four defense-related genes, *KS4, NAC4, PAL1*, and *PAL2*, which are commonly used to detect chitin- or flg22-induced defense responses (Shirsekar et al., 2014), were all significantly induced by chitin or flg22 treatment (Supplementary Figure S2). This result confirmed that treatment of 1-week-old rice seedlings by chitin or flg22 for 3 h can induce strong defense responses. Therefore, we chose this time point for profiling of the ubiquitome.

The experimental workflow for our study is shown in **Figure 1B**. Proteins extracted from 1-week-old rice seedlings treated with water, chitin, or flg22 were subjected to trypsin digestion. We then employed an affinity enrichment strategy, using anti-K-𝜀-GG antibody beads, to generate samples enriched in ubiquitinated peptides. The enriched peptides were detected using LC–MS/MS, and the MS data were then analyzed and the peptides were quantified. To evaluate changes in protein ubiquitination in response to chitin or flg22 treatment, we performed a label-free quantitative proteomic analysis of the lysine ubiquitination substrates. All raw ubiquitome read data presented in this study were deposited in integrated proteome resources (iProX)^5^ with the project ID IPX0001089000.

### Properties of Ubiquitinated Sites, Peptides, and Proteins

Using the above strategy, we identified 2,882 lysine ubiquitination (K^ub^) sites in a total of 1,376 proteins in seedlings treated with water, chitin, or flg22 (Supplementary Figure S3A and Tables S1, S2). Of these, 1,323 K^ub^ sites in 733 proteins were shown to be ubiquitinated in response to chitin, and 1461 K^ub^ sites in 797 proteins were ubiquitinated in response to flg22 (Supplementary Figure S3A and Tables S1, S2). Of the 464 ubiquitinated proteins previously found in young leaves of rice (Xie et al., 2015), 287 (61.9%) were also found in the current study (Supplementary Figure S3B), indicating that the results of this study are reliable. Moreover, more than 70% of the ubiquitinated proteins identified in our study had not been reported previously, i.e., the current results substantially increased the number of known ubiquitinated proteins in rice. The identified lysine ubiquitinated peptides ranged in length from 7 to 66 amino acids. Of the 1,376 ubiquitinated proteins, 9.5% contained 11 amino acids, and 59.3% contained from 8 to 14 amino acids (Supplementary Figure S3C). Among the ubiquitinated proteins, 55% had only one ubiquitinated lysine site, and 19.0, 9.2, 4.9, or 3.3% had two, three, four, or five ubiquitinated sites, respectively (Supplementary Figure S3D and Table S2). In addition, 38 proteins (2.8%) were ubiquitinated at 10 or more lysine sites (Supplementary Figure S3D and Table S2). Eight proteins had more than 15 ubiquitinated sites, including the phenylalanine ammonia-lyase (PAL) PAL1 (26 sites) and PAL5 (17 sites) (Supplementary Figure S3D and Table S2). The following eight significantly enriched motifs from all of the identified ubiquitinated sites were identified using Motif-X analysis (Schwartz and Gygi, 2005): AXXXK^ub^XA, EK^ub^A, AAXXXXK^ub^, K^ub^XA, K^ub^XG, EXXXK^ub^, GK^ub^, and K^ub^XXXA (K^ub^ represents the ubiquitinated lysine, and X represents a random amino acid residue) (Supplementary Figure S3E). Among these, the AAXXXXK^ub^ motif was also found in our previous study of 3-week-old rice seedlings grown in soil without any treatment (Xie et al., 2015). A GO enrichment and KEGG pathway analysis of the ubiquitinated proteins suggested that the rice ubiquitination system regulates diverse cellular processes, such as those involved in primary and secondary metabolism, membrane trafficking, signal transduction, transcription, and translation systems (Supplementary Figures S4, S5 and Table S3), which is consistent with a previous study of the model plant *A. thaliana* (Kim D.Y. et al., 2013).

### Proteomic Analyses Generate a Catalog of Ubiquitination Targets Related to Rice Defense Response

**FIGURE 2:**
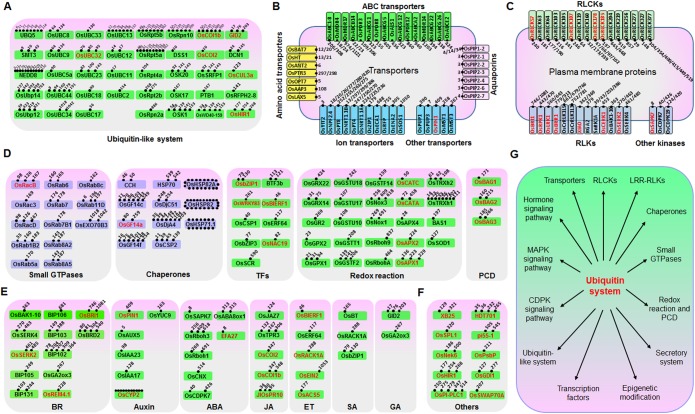
Ubiquitinated proteins involved in many processes in rice seedling response to chitin or flg22. Ubiquitinated proteins were classified to six classes: **(A)** Ubiquitin or ubiquitin-like system proteins; **(B)** Transporters; **(C)** Plasma membrane receptor-like proteins and kinases; **(D)** Defense-related proteins; **(E)** Hormone signaling pathway proteins; **(F)** Other defense-related proteins. **(G)** Model of the regulatory functions of the ubiquitin system in rice immune responses. Proteins that are highlighted in red are reported to be defense-related proteins. For **(A–F)**, ubiquitination sites of each protein are indicated by small spots.

Ubiquitination is known to play important roles in plant defense responses (Marino et al., 2012). We therefore searched for defense-related proteins among the ubiquitinated proteins mentioned above. A total of 185 ubiquitinated proteins that are putatively involved in immune responses were found, of which at least 48 have been reported to be involved in disease responses (Supplementary Table S4). We then classified these 185 proteins into six categories, including “ubiquitin and ubiquitin-like system” (**Figure 2A**), “transporters” (such as amino acids, ABC, aquaporins, ion, and other transporters) (**Figure 2B**), “plasma membrane proteins” (such as receptor like kinase, receptor like cytoplasm kinase, and other kinases) (**Figure 2C**), “proteins involved in signal transduction” (such as small GTPases, chaperones, transcription factors, redox reactions, and programmed cell death) (**Figure 2D**), “hormone pathways” [such as brassinosteroid (BR), auxin, abscisic acid (ABA), JA, ethylene (ET), SA, and gibberellic acid (GA)] (**Figure 2E**), and other proteins that are known to be important for plant defense responses (**Figure 2F**). We identified many previously unreported ubiquitination targets, such as OsSYP22, OsRab1B2, OsRab11D, OsRac7, and OsRacD, which are involved in membrane trafficking; OsAPX1, OsAPX2, OsTRXh1, OsTRXh2, OsGPX1, OsGSTT1, OsRbohA, and OsRbcL1, which are involved in ROS detoxification; and enzymes of the phenylpropanoid metabolic pathway. The data suggested that ubiquitination regulates many aspects of defense responses in rice (**Figure 2G**).

### Chitin- and flg22-Treated Rice Seedlings Have a Similar Ubiquitome

**FIGURE 3:**
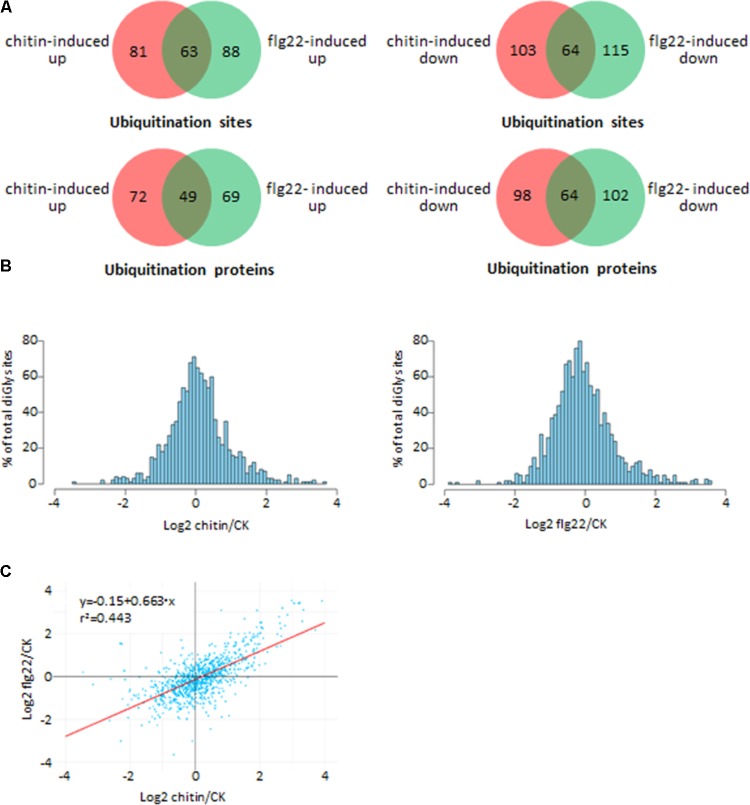
Comparison of the ubiquitinated proteins from rice seedlings treated with chitin or flg22. **(A)** Venn diagram showing the overlap between ubiquitination sites and proteins induced or suppressed by chitin or flg22. **(B)** Histograms depicting the log2(L/H) ratios for all quantified diGly sites in the three chitin-treated replicates (left) and three flg22-treated replicates (right). **(C)** Correlation of the ubiquitomes of chitin- or flg22-treated samples.

We investigated differences in the rice ubiquitome after treatments with chitin, flg22, or water (control). A total of 1,323 quantifiable ubiquitinated sites were identified in the chitin-treated seedlings, and 1,461 quantifiable ubiquitinated sites were identified in the flg22-treated seedlings (Supplementary Tables S5, S6). We used ratios of ≥2.0 and ≤0.5 with a *t*-test *P* < 0.05 as the thresholds for increase or decrease in abundance, respectively. In the chitin-treated samples, 144 ubiquitination sites in 121 proteins had increased ubiquitination levels, and 167 sites in 162 proteins had decreased ubiquitination levels. In the flg22-treated samples, 151 ubiquitination sites in 118 proteins had increased ubiquitination levels, and 179 sites in 166 proteins had decreased ubiquitination levels (**Figures 3A,B**; Supplementary Tables S5–S8). We then searched for an overlap between the chitin-responsive and flg22-responsive ubiquitome. In both chitin- and flg22-treated seedlings, 63 sites in 49 proteins and 64 sites in 64 proteins showed increased and decreased ubiquitination, respectively (**Figure 3A** and Supplementary Tables S7, S8). The ratios of the chitin-responsive and flg22-responsive ubiquitomes were compared with a scatter diagram (**Figure 3C**). The analysis demonstrated that many ubiquitinated proteins have a similar pattern of increasing or decreasing of ubiquitination levels to chitin and flg22 treatments.

### GO Analyses of Ubiquitinated Targets in Chitin- or flg22-Treated Rice Seedlings

**FIGURE 4:**
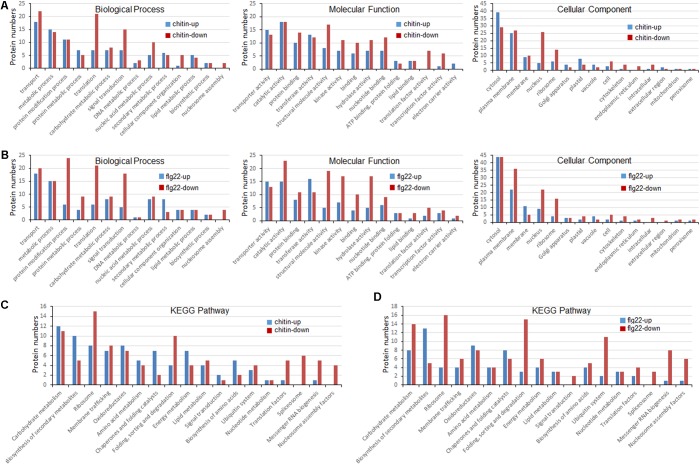
Gene ontology (GO) and Kyoto Encyclopedia of Genes and Genomes (KEGG) analyses of ubiquitinated proteins in response to chitin and flg22. **(A)** GO classification of the ubiquitinated proteins induced by chitin. **(B)** GO classification of the ubiquitinated proteins induced by flg22. **(C)** KEGG pathway enrichment of the ubiquitinated proteins induced by chitin. **(D)** KEGG pathway enrichment of the ubiquitinated proteins induced flg22.

To better understand the lysine ubiquitome changes in rice seedlings induced by chitin or flg22, we performed a GO functional annotation analysis (**Figures 4A,B**). For both treatments, biological process analysis of the GO functional clustering indicated that proteins with increased ubiquitination were associated with “transport,” “metabolic process,” “protein metabolism,” “protein modification process,” “translation,” “carbohydrate metabolic process,” and “signal transduction.” It is noteworthy that the decreased ubiquitination proteins involved in “protein modification process,” “translation,” “signal transduction,” “nucleic acid metabolic processes,” and “nucleosome assembly” were significantly enriched compared to the increased ones. In the molecular function category, the ubiquitination levels of proteins involved in “transporter activity,” “catalytic activity,” “protein binding activity,” “transferase activity,” “structural molecule activity,” and “kinase activity” were significantly increased, while the ubiquitination levels of proteins involved in “catalytic activity,” “protein binding activity,” “structural molecule activity,” “kinase activity,” “hydrolase activity,” and “nucleotide binding activity” were severely decreased. In the cellular compartment category, proteins undergoing significant changes were mostly annotated as localized in the cytosol, plasma membrane, membrane, ribosome, and nucleus. The ubiquitination levels of proteins localized in the plasma membrane, ribosome, and nucleus were significantly decreased in response to the PAMP treatment. Overall, these results indicated that ubiquitination levels of proteins involved in translation and nuclear processes were induced by the elicitor treatments.

To identify the cellular pathways affected by the chitin or flg22 treatments, we performed a pathway clustering analysis using KEGG (**Figures 4C,D**). The results indicated that “ribosome biogenesis”; “protein folding, sorting, and degrading”; “messenger RNA biogenesis”; and “nucleosome assembly” were the most prominent pathways enriched in proteins with down-regulated ubiquitination, suggesting that the elicitors function as regulatory factors in protein biosynthesis. In contrast, “carbohydrate metabolism,” “biogenesis of secondary metabolites,” “ribosome,” “membrane trafficking,” “oxidoreductases,” “amino acid metabolism,” “chaperones and folding catalysis,” and “energy metabolism” pathways were the most prominent pathways enriched in proteins with up-regulated ubiquitination. The results suggested that similar pathways were enriched in proteins with down- and up-regulated ubiquitination levels in chitin-treated vs. flg22-treated rice seedlings, consistent with the inference that the two elicitors act through similar pathways in the induction of plant defense responses.

### Protein Interaction Networks for the Chitin- or flg22-Triggered Ubiquitome

**FIGURE 5:**
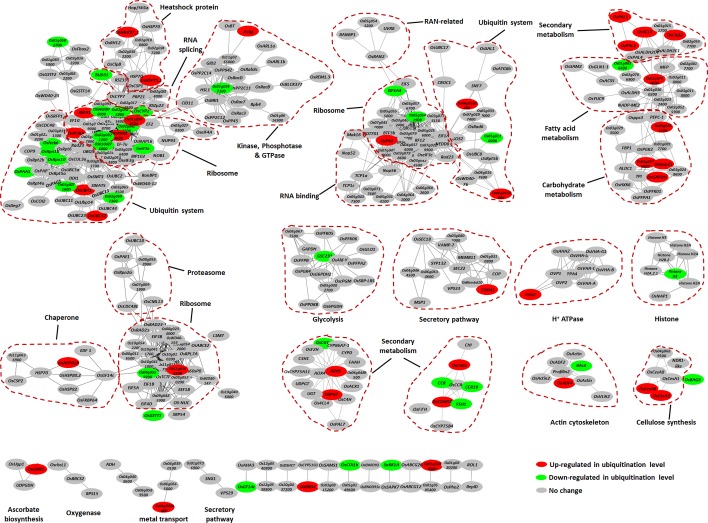
Protein interaction network of the ubiquitinated proteins. Protein interaction networks were generated with the complete list of ubiquitinated proteins using the STRING database and were visualized using the Cytoscape program. The node colors reflect ubiquitinated level up-regulated (≥2-fold, red) or down-regulated (≤2-fold, green) by chitin or flg22 treatment. Important clusters are surrounded by dashed lines.

To further elucidate the significance and extent of ubiquitination in response to the chitin or flg22 treatments, we generated an interaction network using the Search Tool for Retrieval of Interacting Genes/Proteins (STRING) database (Szklarczyk et al., 2015). A number of sub-networks were identified, including “ubiquitin system,” “ribosome,” “RNA splicing,” “protein kinase and phosphatase,” “small GTPase,” “chaperone,” “RNA binding protein,” “secondary metabolism,” “fatty acid metabolism,” “carbohydrate metabolism,” “proteasome,” “glycolysis,” and “histone” (**Figure 5**).

The most abundant ubiquitinated proteins in the ubiquitome network was ribosome component, which was present in several sub-networks. When elicited by the PAMPs, some of the ribosome proteins showed increased ubiquitination, while others showed decreased ubiquitination (**Figure 5**), suggesting that the ubiquitination system can regulate the protein translation during defense responses by either increasing or decreasing protein abundance. Similarly, the ubiquitination levels of different proteins in the ubiquitin system either increased or decreased in response to PAMPs (**Figure 5**), indicating that the ubiquitin system itself shows complex regulation during defense responses.

We found that components of several secondary metabolism sub-networks had greatly elevated ubiquitination levels in response to both elicitors. These proteins included OsPAL1, OsPAL2, Os4CL3, OsCHS1, OsCOMT1, OsCAD2, XDH1, and UDPGT, which are predicted to be involved in the phenylpropanoid biosynthetic pathway and purine metabolism (**Figure 5**). A number of heat shock proteins were prominent in the ubiquitome network, of which OsMed37_1, OsHSP71.1, and OsHSP82A showed elevated ubiquitination levels in response to both chitin and flg22 (**Figure 5**). Proteins involved in cellulose synthesis (OsCesA3 and OsCesA6), plasma membrane H^+^ ATPase (OSA7), and secretory pathway (COPA1) also showed induced ubiquitination levels (**Figure 5**). Finally, many glycolysis enzymes and histone proteins were also present in the network, some of which (GSC233 and Histone H3) had down-regulated ubiquitination in response to PAMPs (**Figure 5**).

In summary, these data indicated that chaperones (especially the heat shock proteins) and proteins in secondary metabolism, carbohydrate metabolism, secretory pathway, cellulose synthesis, and H^+^ ATPase sub-networks had increased ubiquitination levels, while proteins in the glycolysis and histone mainly had decreased ubiquitination levels.

### Ubiquitination Affects Many Processes Involved in PTI in Chitin- and flg22-Treated Rice Seedlings

**FIGURE 6:**
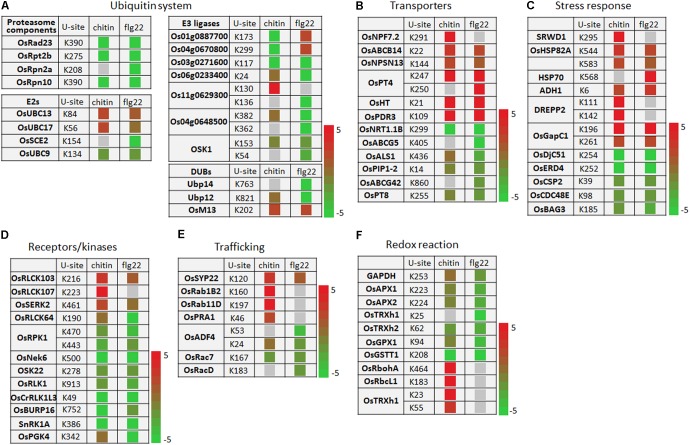
Ubiquitination events of key proteins of cellular defense processes in response to chitin and flg22 treatments. **(A–F)** Key proteins with changes in ubiquitination levels involved in **(A)** the ubiquitin system; **(B)** transporters; **(C)** stress response; **(D)** receptors/kinases; **(E)** trafficking; and **(F)** redox reaction. U-site, the lysine residue linked by ubiquitin.

The ubiquitination levels of many ubiquitination system-related proteins changed in response to the PAMP treatments. These included the 26S protease regulatory subunit OsRpt2b and the 26S proteasome non-ATPase regulatory subunits OsRpn2a and OsRpn10 (**Figure 6A**). The ubiquitin-conjugating enzymes OsUBC13 and OsUBC17 had up-regulated ubiquitination levels. The SUMOylation pathway ubiquitin conjugating enzymes OsSCE2 and OsUBC9 had reduced levels of ubiquitination, suggesting that the sumoylation system may interact with the ubiquitination system during PTI. The ubiquitination levels of several E3 ligases were increased or decreased by responding to chitin or flg22; some E3 ligases were decreased by responding to chitin, while others were decreased by responding to flg22, and Os03g0271600 was decreased by responding to both PAMPs (**Figure 6A**).

The ubiquitination levels of numerous transporters were increased or decreased by responding to the chitin or flg22 treatments. Transporters increased in ubiquitination included OsNPF7.2, OsABCB14, OsNPSN13, OsPT4, OsHT, and OsPDR3, while those decreased in ubiquitination included OsNRT1.1B, OsABCG5, OsALS1, OsPIP1-2, OsABCG42, and OsPT8 (**Figure 6B**). A number of ubiquitinated stress-responding proteins was also increased or decreased by responding to chitin or flg22 (**Figure 6C**), suggested that some transporters and stress-responsive proteins could be activated during defense responses, while others could be repressed or targeted for degradation by the ubiquitination system. We also found that both chitin and flg22 treatment resulted in elevated or repressed ubiquitination levels of similar transporter and stress-responding proteins. The ubiquitination of one PCD-related protein, OsBAG3, was decreased in ubiquitination by responding to both of the chitin and flg22 treatments.

The activity and stability of plant receptor-like proteins must be tightly regulated to ensure appropriate defense activation and to avoid adverse effects on growth and development. The ubiquitin-proteasome system (UPS)-mediated proteolysis plays a key role in this process (Furlan et al., 2012). Ubiquitination levels of the receptors OsRLCK103, OsRLCK107, and OsSERK2 were up-regulated by chitin and/or flg22, while the ubiquitination levels of several receptors or protein kinases, including OsRLCK64, OsRPK1, OsNek6, OSK22, OsRLK1, OsCrRLK1L3, OsBURP16, SnRK1A, and OsPGK4, were decreased in ubiquitination by responding to chitin or flg22 treatments (**Figure 6D**). Membrane trafficking of receptors depends on accurate targeting of transport vesicles between precisely defined membrane-bound compartments along the biosynthetic and endocytic pathways (Furlan et al., 2012). Some membrane trafficking proteins, such as OsSYP22, OsRab1B2, OsRab11D, and OsPRA1, had increased ubiquitination levels upon chitin or flg22 induction, while OsADF4, OsRac7, and OsRacD had decreased ubiquitination upon flg22 induction (**Figure 6E**).

The ubiquitination levels of proteins related to ROS production or detoxification, such as GAPDH, OsAPX1, OsAPX2, OsTRXh2, OsGPX1, and OsGSTT1, were clearly decreased in ubiquitination by responding to chitin or flg22 treatments (**Figure 6F**). Interestingly, OsRbohA and OsRbcL1 had up-regulated ubiquitination levels when induced by chitin but lacked ubiquitination in the flg22-treated samples. The OsTRXh1 ubiquitination level was decreased in ubiquitination in flg22-treated samples but increased in chitin-treated samples. These data indicated that the ubiquitomes activated by chitin and flg22 are generally similar but have differences.

### Roles of Ubiquitination in Hormone-Mediated Defense Signaling in Chitin- and flg22-Treated Rice Seedlings

**FIGURE 7:**
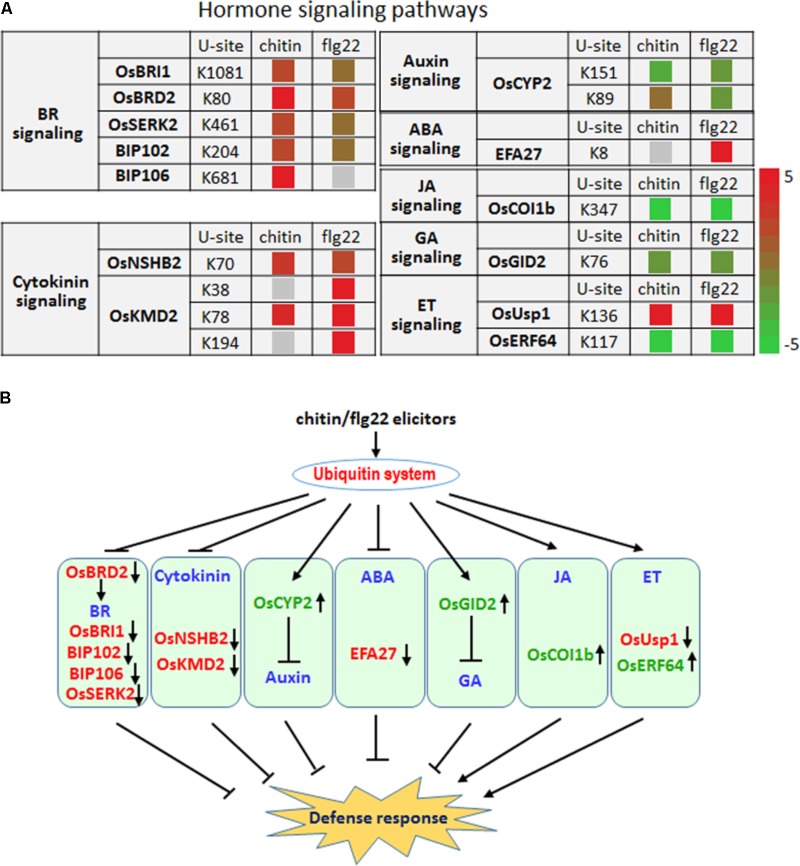
The ubiquitin system coordinates hormone signaling pathways, metabolite biosynthesis, and developmental processes upon elicitor treatment. **(A)** Changes in the ubiquitination levels at the indicated sites for proteins involved in hormone signaling pathways. **(B)** Regulatory functions of the ubiquitin system in hormone signaling pathways.

Plant hormones play essential roles in fine-tuning rice defense responses in response to pathogen attack (Bari and Jones, 2009). Our quantitative proteomic analysis identified hormone signaling-related proteins whose ubiquitination level changed more than twofold following chitin or flg22 treatment (**Figure 7A**).

Six proteins that are involved in the brassinosteroid signaling pathway, OsBRI1, OsBRD2, OsSERK2, BIP102, and BIP106, had significantly higher ubiquitination levels after treatment with either elicitor (**Figure 7A**), indicating that the ubiquitination system represses the BR signaling pathway as part of a defense response. The ubiquitination level at K89 and K151 of the auxin pathway protein OsCYP2, which has been shown to degrade AUX/IAA proteins (Zheng et al., 2013), was reduced by chitin or flg22 treatment (**Figure 7A**). Because auxin is generally a suppressor of innate immunity in rice, the decreased ubiquitination level of OsCYP2 suggested that the defense response could be activated and that the auxin signaling pathway in rice seedlings could be repressed by chitin or flg22 treatment. A member of the rice KmD family of proteins, OsKMD2, has been reported to regulate the transcriptional response to cytokinin (Kim H.J. et al., 2013), and OsNSHB2, which is a hemoglobin (HB), is known to be induced by cytokinin. HBs can contribute to oxygen storage and transport, nitric oxide detoxification, and oxygen diffusion (Lira-Ruan et al., 2011). The ubiquitination level of both OsNSHB2 and OsKMD2 was increased by responding to chitin and flg22 (**Figure 7A**), suggesting that the cytokinin signaling pathway could be repressed by these elicitors.

We observed that ubiquitination of the key factor OsCOI1b in JA signaling was not detected after the chitin or flg22 treatment. OsCOI1b, part of the E3 ubiquitin ligase complex, interacts with JAZ proteins and targets them for degradation in response to JA signaling. Following application of chitin or flg22, decreased in ubiquitination could lead to increased OsCOI1b protein levels, potentially resulting in enhanced JA signaling (**Figure 7A**). The ubiquitination level of two ET signaling pathway proteins, OsUsp1 and OsERF64, also changed following chitin and flg22 treatment (**Figure 7A**). The ET-responding transcription factor OsERF64 was not detected in chitin and flg22 treated samples, while the ET-responding stress adaptive protein OsUSP1 had increased ubiquitination level upon both treatment. The ABA-responsive protein EFA27 was increased in ubiquitination level by responding to flg22 but not detected in chitin-treated samples (**Figure 7A**). GID2 is involved in the GA signaling pathway, and its ubiquitination levels decreased approximately twofold in response to chitin or flg22 treatment (**Figure 7A**). This could lead to elevated GID2 protein levels and promote its function as an F-box subunit of the SCF E3 complex, regulating the GA-dependent degradation of SLR1 (Gomi et al., 2004). Although SA is important in rice basal defense, we did not identify any SA-related components with changes in ubiquitination levels. Taken together, the quantitative proteomic analysis indicated that multiple hormone signaling pathways were influenced by the ubiquitination system in response to treatment with the PAMPs chitin or flg22 (**Figure 7B**).

### Enzymes in the Phenylpropanoid Pathway Are Targeted by the Ubiquitination System After PAMP Treatments

**FIGURE 8:**
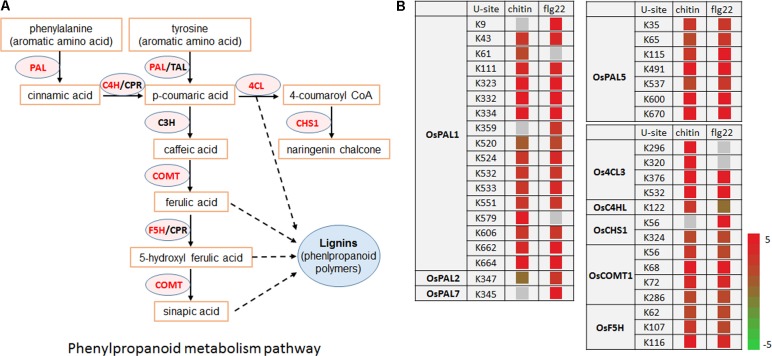
Ubiquitinated proteins during defense responses include enzymes in the phenylpropanoid metabolic pathway. **(A)** Schematic diagram of the phenylpropanoid metabolic pathway and its key enzymes. **(B)** Ubiquitination sites in proteins encoding enzymes in the phenylpropanoid metabolic pathway.

Lignin and other compounds synthesized through the phenylpropanoid pathway are important in responses to environmental stresses and pathogen infection (Fraser and Chapple, 2011). Interestingly, we found that many enzymes in the phenylpropanoid metabolic pathway, including OsPAL1, OsPAL2, OsPAL7, OsPAL5, Os4CL3, OsC4HL, OsCHS1, OsCOMT1, and OsF5H, had significantly increased ubiquitination levels in response to the chitin or flg22 treatment. Among them, OsPAL1, OsPAL5, Os4CL3, and OsCOMT1 contained multiple ubiquitination sites and their ubiquitination levels were significantly induced by both treatments (**Figures 8A,B**). These data are consistent with the finding that the phenylpropanoid metabolic pathway in rice was repressed 3 h after chitin or flg22 treatment.

## Discussion

Although many reports have described specific examples of ubiquitination in plant defense responses, our understanding on global changes in the plant ubiquitome is still lacking. In this study, we combined the high-resolution LC–MS/MS with an affinity enrichment method to enhance ubiquitinated peptides, allowing the large scale and site-specific quantification of ubiquitination in rice seedlings in response to two PAMPs. We identified many new proteins targeted by elicitor-induced ubiquitination, highlighting the complexity of the regulatory system in plant defense responses.

### Common and Different Effects of Ubiquitination Elicited by Chitin and flg22

Previous studies have revealed that the gene expression patterns elicited by different PAMPs are largely similar during the late stages of defense responses, and that different PAMPs can induce similar plant signaling pathways and cellular responses (Boller and Felix, 2009). In this study, we compared the ubiquitome data obtained from samples treated with chitin or flg22 for 3 h, which is a late-defense response time point, and also found that the changes in ubiquitination levels elicited by these two PAMPs were broadly similar. These results suggest that the downstream signaling pathways and cellular processes involved in defense responses are also similar at the post-translational modification level. However, further studies are needed to determine the ubiquitome changes elicited by different PAMPs during the early stages of the defense response.

We did observe some differences between chitin- and flg22-regulated ubiquitination, including ubiquitination-associated with E3 ligases (**Figure 6A**), ROS production or detoxification (**Figure 6F**), and hormone pathways (such as ABA signaling) (**Figure 7A**). These data suggested that differences in these processes still exist between chitin- and flg22-regulated ubiquitination during the late stages of plant defense responses. These differences might have several explanations. For example, some of these ubiquitinated proteins could be specific to different elicitors or the responding times to different elicitors could be different. In *Arabidopsis*, ROS accumulation triggered by flg22 is controlled by RbohD (Nühse et al., 2007), while in *Nicotiana benthamiana* triggered by fungal cell wall extracts requires NbRbohA and NbRbohB (Yoshioka et al., 2003). In rice, OsRbohA is involved in rapid ROS production, while OsRbohE is involved in late ROS production during the immune response to *Acidovorax avenae* (Yoshiaki et al., 2005). In this study, we found that the ubiquitination level of OsRbohA is up-regulated, which may lead to its degradation. It was reported that phosphorylation patterns in RbohD also differed following treatment with flg22 and xylanase in *Arabidopsis*, although both elicitors induce RbohD phosphorylation at similar locations (Benschop et al., 2007). Interestingly, the RBOH proteins belongs to the seven transmembrane proteins, which could either be poly-ubiquitinated for lysosomal or proteasomal degradation, or mono-ubiquitinated for endocytosis internalization (Shenoy, 2007). Which ubiquitination type(s) of the RBOH proteins could be undergo and what the subsequent functions could be, both are intriguing questions. The ubiquitination-mediated regulation of E3 ligases that specifically respond to different elicitors or pathogens has also been reported, especially targeting the receptors (Furlan et al., 2012). For example, the rice E3 ligase XB3 can respond to *Xanthomonas oryzae* pv. *oryzae* and interact with the LRR-RLK XA21 to affect its accumulation (Wang et al., 2006). The *Medicago truncatula* E3 ligase PUB1 can respond to *Sinorhizobium meliloti* and negatively regulate nodulation (Mbengue et al., 2010).

### Ubiquitination Up- and Down-Regulates Plant Defense-Related Protein Levels

After chitin or flg22 treatment, a similar proportion of ubiquitination sites were up-regulated or down-regulated. Proteins with increased ubiquitination levels may themselves be degraded by the ubiquitination system, and in this case acts to reduce protein abundance. Proteins with reduced ubiquitination levels may avoid degradation, and in this case acts to increase protein abundance. The ubiquitination system can therefore rapidly enhance or suppress different cellular processes to facilitate defense responses. For example, upon elicitor induction, the defense-related receptors were mostly decreased in ubiquitination levels, while the phenylpropanoid metabolic pathway could be suppressed by ubiquitination. Similarly, the hormone signaling pathways were activated while the BR and cytokinin pathways were repressed. Surprisingly, the auxin and GA signaling pathways were both repressed, likely through decreases in the ubiquitination levels of OsCYP2 and OsGID2, which are key proteins in the respective signaling pathways (**Figure 7A**).

### Key Processes in Plant Defense Responses Are Modified by Ubiquitination

The activity and stability of plant plasma membrane receptors, as well as protein kinases, are tightly regulated to ensure appropriate defense responses (Antolin-Llovera et al., 2012). There is growing evidence that the ubiquitination system can modulate the abundance of plasma membrane receptors by ubiquitination of the receptors themselves or of membrane trafficking components or receptor recycling components (Furlan et al., 2012). However, the detailed mechanisms of this ubiquitination-mediated regulation remain unknown. We observed that the ubiquitination levels of four receptors, OsRLCK103, OsRLCK107, OsSERK2, and a BAK1 like protein, were significantly up-regulated by chitin or flg22 treatment. In contrast, ubiquitination levels were down-regulated for nine receptors or protein kinases. Understanding how the ubiquitination of these proteins is regulated will help reveal how the ubiquitination of plasma membrane receptors or protein kinases contributes to plant immunity. Researchers recently found, for example, that receptor OsRLCK176 is negatively regulated by OsCPK4 and that activation of OsCPK4-OsRLCK176 phosphorylation circuit can suppress OsRLCK176 ubiquitination to coordinate plant immunity (Wang et al., 2017).

When attacked by pathogens, ROS rapidly accumulate as part of the defense responses, while ROS-detoxifying enzymes are synthesized to balance the cellular redox changes (Mendoza, 2011). Cellular redox homeostasis changes rapidly, and dynamic changes in the protein levels involved in ROS production and ROS-detoxifying enzymes are essential for responses to biotic or abiotic stresses. Ubiquitin-mediated protein degradation allows the rapid regulation of protein levels, and we have identified a large number of proteins involved in ROS production or detoxification and that have significantly down-regulated ubiquitination following chitin or flg22 treatment.

### Ubiquitination of Phenylpropanoid Pathway Enzymes Is Significantly Elevated During Rice Defense Responses

Our results revealed that regulation of the phenylpropanoid pathway by ubiquitination is associated with plant innate immunity. Phenylpropanoid biosynthesis converts L-phenylalanine into a range of aromatic metabolites that act as antioxidants, contributing to detoxification, or that act as signaling molecules to mediate defense responses (Fraser and Chapple, 2011). Multiple mechanisms have been reported to control the activity of the phenylpropanoid pathway rate-limiting enzyme PAL (Vogt, 2010). PAL activity has also been shown to be induced transiently in response to biotic or abiotic stimuli as an early defense response by *de novo* mRNA synthesis and at the transcriptional level (Edwards et al., 1985; Liang et al., 1989). Other phenylpropanoid biosynthetic genes are also regulated at the transcriptional level in response to biotic and abiotic stress (Liang et al., 1989). Recently, several phenylpropanoid pathway enzymes, including AtPAL-1, -2, and -3, were found to be potentially ubiquitinated in *A. thaliana* (Kim D.Y. et al., 2013), and (poly)ubiquitination of four *A. thaliana* PAL isoforms was confirmed by immunoblot analysis (Zhang et al., 2013). In addition to OsPAL1, OsPAL2, OsPAL5, and OsPAL7, we found that the ubiquitination levels of other phenylpropanoid pathway enzymes, including Os4CL3, OsC4HL, OsCHS1, OsCOMT1, and OsF5H, were significantly greater after 3 h stimulation with chitin or flg22. Whether these proteins are degraded by the ubiquitin-26S proteasome system in response to pathogen infection remains to be determined.

## Conclusion

In summary, the systematic identification of ubiquitination targets in rice plants treated by PAMPs revealed the diversity and complexity of the defense responses involving the ubiquitination system. The ubiquitination system likely plays a critical role in fine-tuning PTI. The large pool of ubiquitination targets identified here will serve as a valuable resource for understanding how the ubiquitination system regulates defense responses upon pathogen attack.

## Author Contributions

X-LC performed most of the experiments, data processing, and bioinformatics analyses. XX performed the western blot analyses. LW and CL prepared the samples for protein sequencing. WL, G-LW, and X-LC designed the experiments. X-LC, WL, G-LW, LZ, XZ, and FL wrote the manuscript.

## Conflict of Interest Statement

The authors declare that the research was conducted in the absence of any commercial or financial relationships that could be construed as a potential conflict of interest.

## Footnotes

1 http://rice.plantbiology.msu.edu/index.shtml

2 http://www.ebi.ac.uk/GOA/

3 http://www.genome.jp/tools/kaas/

4 https://string-db.org/

5 http://www.iprox.cn
