# Supplementary material for: Proteomic Analysis of Ubiquitinated Proteins in Rice (Oryza sativa) After Treatment With Pathogen-Associated Molecular Pattern (PAMP) Elicitors
Source: Front Plant Sci. 2018 Jul 23;9:1064. doi: 10.3389/fpls.2018.01064 (PMC6064729; doi:10.3389/fpls.2018.01064)
Supplement: Supplementary file 10 [file Image_1.PDF]

Supplementary Materials

Proteomic analysis of ubiquitinated proteins in rice (*Oryza sativa*) after treatment with pathogen-associated molecular pattern (PAMP) elicitors

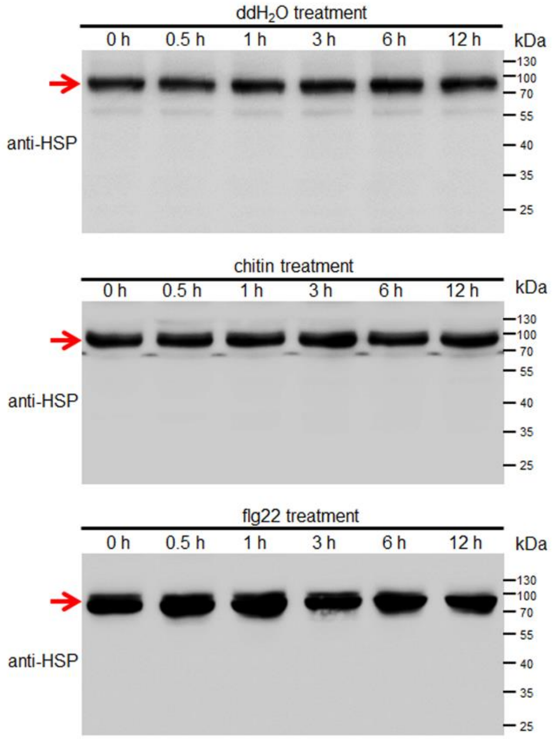

Figure S1 | Loading amount evaluation of total proteins by western blot analysis using anti-HSP. Protein extracts (20 μg) of indicated samples were separated by 12% SDS-PAGE and subjected to Western blot analysis. Arrows indicate putative HSP90 proteins detected by anti-HSP antibody.

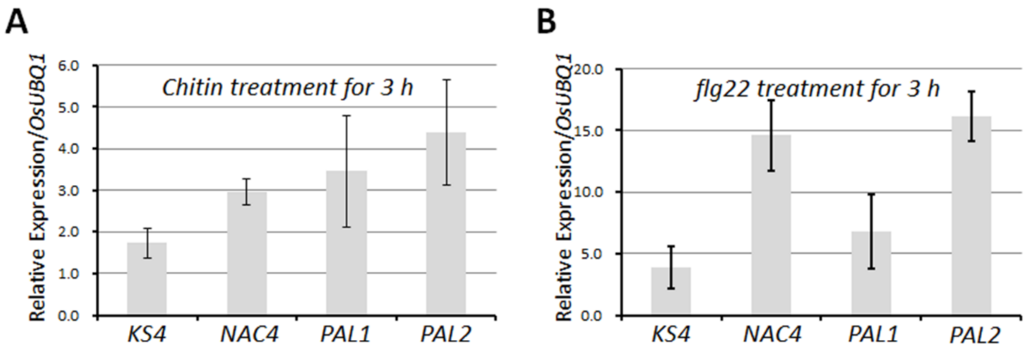

Figure S2 | Expression changes of defense-related genes in response to chitin or flg22. **(A)** Expression changes of some rice defense-related genes induced by chitin. **(B)** Expression changes of some rice defense-related genes induced by flg22. For **(A)** and **(B)**, the one-week old rice seedlings were induced by adding 8 nmol/l chitin and 1 μmol/l flg22 for 3 h for treatment.

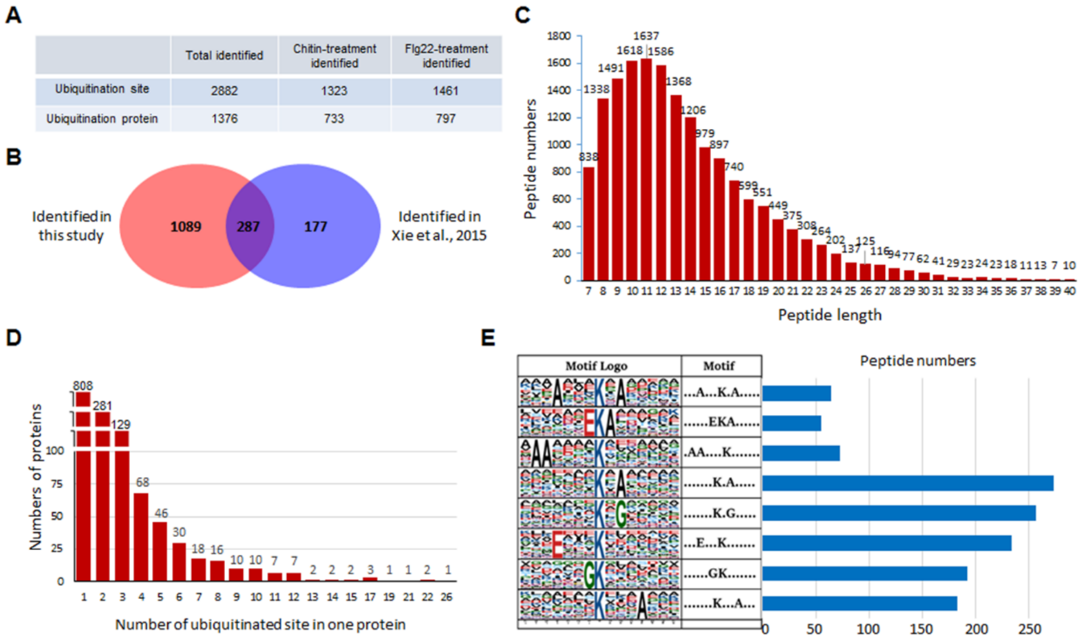

Figure S3 | Properties of ubiquitinated sites, peptides and proteins. **(A)** Summary statistics for the ubiquitome analysis. **(B)** Comparing of rice ubiquitinated proteins identified in this study and in a former report (Xie *et al.*, 2015). **(C)** Distribution of ubiquitinated peptides based on their length. **(D)** Distribution of ubiquitinated proteins based on the number of modified sites. **(E)** Ubiquitination motifs and conservation of ubiquitination sites were analyzed using the Motif-X algorithm. Numbers of ubiquitinated peptides containing each motif are indicated.

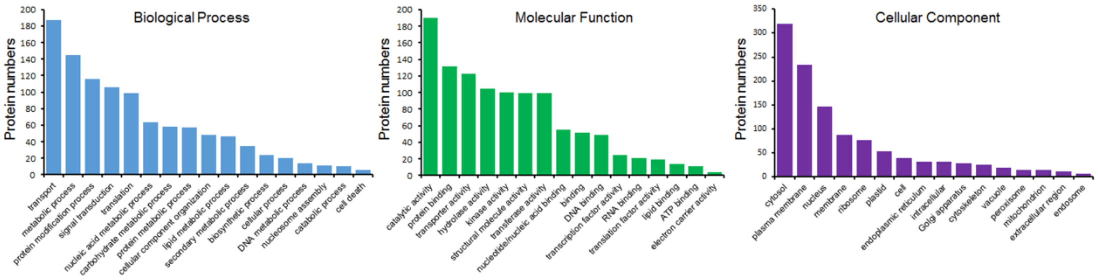

Figure S4 | Gene ontology (GO) classification of all the ubiquitinated proteins identified in this study.

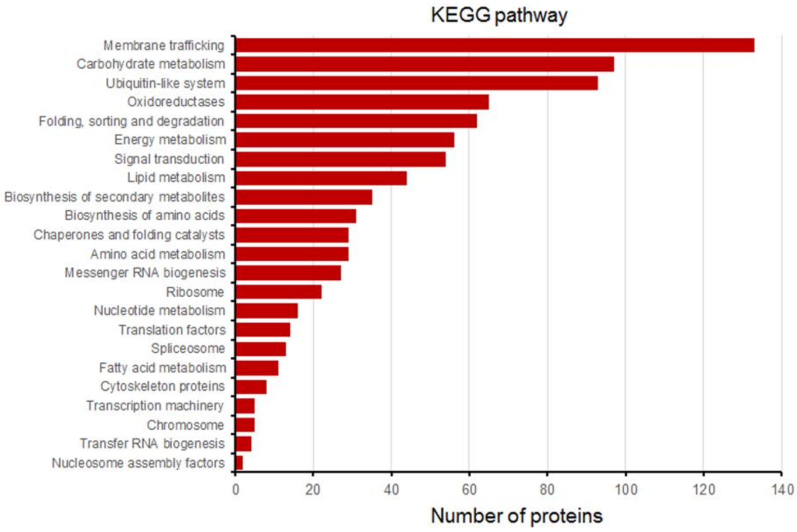

Figure S5 | Kyoto Encyclopedia of Genes and Genomes (KEGG) pathway enrichment of all the ubiquitinated proteins identified in this study.
